# When Guidelines Meet Reality: The Combined Impact of Assay Variability and Prescribing Differences on TSH Management in Thyroid Cancer

**DOI:** 10.3390/cancers17243912

**Published:** 2025-12-07

**Authors:** Petra Petranović Ovčariček, Alfredo Campennì, Federica D’Aurizio, Mauro Imperiali, Angela Alibrandi, Rosaria Maddalena Ruggeri, Lilla Bonanno, Luca Giovanella

**Affiliations:** 1 Department of Oncology and Nuclear Medicine, University Hospital Center Sestre Milosrdnice, 10000 Zagreb, Croatia; 2 School of Medicine, University of Zagreb, 10000 Zagreb, Croatia; 3 Nuclear Medicine Unit, Biomedical and Dental Sciences and Morpho-Functional Imaging, University of Messina, 98124 Messina, Italy; acampenni@unime.it; 4 Laboratory for Clinical Pathology, Department of Laboratory Medicine, University Hospital of Udine, 33100 Udine, Italy; federica.daurizio@asufc.sanita.fvg.it; 5 Labor Dr Risch, Sonic Switzerland, 6900 Lugano, Switzerland; mauro.imperiali@risch.ch; 6 Department of Economics, University of Messina, 98125 Messina, Italy; aalibrandi@unime.it; 7 Endocrinology Unit, Department of Clinical and Experimental Medicine, University of Messina, 98125 Messina, Italy; rosaria.ruggeri@unime.it; 8 IRCCS Centro Neurolesi “Bonino Pulejo”, 98125 Messina, Switzerland; lilla.bonanno@irccsme.it; 9 Nuclear Medicine and Thyroid Center, Gruppo Ospedaliero Moncucco, 6900 Lugano, Switzerland; 10 Nuclear Medicine and Thyroid Center, University Hospital of Zurich, 8091 Zurich, Switzerland

**Keywords:** thyroid-stimulating hormone, immunoassay, differentiated thyroid cancer, response to treatment, risk stratification, levothyroxine

## Abstract

Background/Objectives: Patients with differentiated thyroid cancer (DTC) receive thyroxine substitution targeting thyroid-stimulating hormone (TSH) levels based on their treatment response category. However, variations in prescribing and inter-assay TSH variability may result in over or undertreatment. Methods: We measured TSH in 220 consecutive DTC patients using three automated immunoassay platforms (Elecsys, Atellica, Alinity). Each patient was assigned to a response-to-therapy category (Excellent Response [ER], Indeterminate Response [IndR], Biochemical Incomplete Response [BIR], Structural Incomplete Response [SIR]) by an experienced thyroid oncologist. We defined recommended TSH targets according to the American Thyroid Association (ATA) 2015 guidelines and the response-adapted ATA 2025 framework that allows progressive relaxation of TSH suppression in patients with ER while maintaining tight suppression in those with persistent disease. Analytical agreement between assays was assessed using Passing–Bablok regression and Bland–Altman analysis. Clinical appropriateness was evaluated by classifying each measured TSH value as below, within, or above the recommended range for that patient’s response category. Results: The three immunoassays demonstrated high analytical agreement with only minor biases unlikely to affect clinical interpretation. However, significant deviations from guideline-defined TSH targets were observed. Among ER patients, 37% remained oversuppressed despite the absence of active disease. Conversely, in IndR or BIR patients, 76% had TSH levels above the recommended range, indicating undersuppression where residual disease could not be excluded. SIR patients were generally managed appropriately. The ATA 2025 framework reclassified more ER patients as appropriately managed, but undersuppression persisted in non-ER patients. Conclusions: Guidelines are not uniformly applied in thyroxine dosing for DTC patients. TSH immunoassays have achieved adequate analytical performance. The focus must now shift toward addressing clinical, educational, and systemic factors that prevent optimal levothyroxine management.

## Simple Summary

Patients treated for thyroid cancer require thyroid hormone replacement therapy to keep their thyroid-stimulating hormone levels at specific targets based on whether they have active disease. We investigated whether differences between laboratory tests and variations in how physicians prescribe medication might lead to inappropriate treatment. We measured thyroid-stimulating hormone levels in 220 patients with thyroid cancer using three different laboratory platforms and compared the results with treatment guidelines. We found that the laboratory tests were highly consistent with each other. Still, many patients received inappropriate doses of thyroid hormone therapy—some cancer-free patients were overtreated, while others with possible remaining disease were undertreated. Our findings demonstrate that modern laboratory tests are sufficiently accurate for patient monitoring, and the focus should now shift toward improving how physicians prescribe thyroid hormone therapy to ensure patients receive optimal treatment that balances cancer control with quality of life.

## 1. Introduction

Differentiated thyroid cancer is the most prevalent endocrine malignancy, with generally favorable outcomes following surgical and, where indicated, radioiodine treatment [1,2,3,4,5,6,7,8,9,10,11,12,13,14,15,16,17,18,19,20,21]. Notably, residual or metastatic DTC cells may retain TSH receptors, and suppressing TSH can reduce the stimulation of any remaining thyroid cancer cells, potentially reducing the risk of recurrence and improving disease-free survival [22]. Thus, modulation of TSH levels through levothyroxine therapy is a cornerstone of DTC management. For a long time, most DTC patients underwent long-term TSH suppression (i.e., <0.1 mIU/L); however, TSH suppression beyond low–normal levels has not demonstrated clear recurrence reduction in low-risk patients with excellent response (i.e., the large majority of DTC patients nowadays) and potential adverse effects of prolonged TSH suppression, including atrial fibrillation, heart failure, decreased bone mineral density and symptoms of thyroid overactivity, require careful consideration [23]. Accordingly, TSH measurement is the cornerstone test for assessing appropriate levothyroxine dosing and avoiding inappropriate TSH suppression or elevation, respectively. However, although current TSH immunometric assays are standardized against the 2nd International Standard (IS) - WHO 80/558, concerns about inter-assay variability persist, especially since TSH values influence the long-term intensity of thyroxine treatment [24]. Differences in antibody configuration, calibration, and detection systems may lead to inconsistencies in TSH measurement across assay platforms, potentially altering treatment decisions. The 2015 American Thyroid Association (ATA) guidelines recommended tailored TSH suppression after initial treatment for DTC, based on the patient’s risk category: high risk [TSH ≤ 0.1 mIU/L], intermediate risk [TSH 0.1–0.5 mIU/L], and low risk [TSH 0.5–2 mIU/L]. Furthermore, TSH values should be re-titrated during follow-up according to the patient’s dynamic response to therapy. Specifically, TSH levels between 0.5 and 2 mIU/L and 0.1–0.5 mIU/L were recommended for patients with ER and those with BIR or IndR, respectively, whereas levels ≤ 0.1 mIU/L were advised in patients with SIR (Table 1) [25]. Although these criteria remain the foundation of TSH modulation, achieving and maintaining target TSH levels in real-world practice remains challenging, as shown by many cohort studies and meta-analyses [26]. Finally, the 2025 ATA guidelines, released in October 2025, have introduced relevant updates. First, instead of fixed numerical cutoffs, the recommended TSH targets are now expressed relative to the assay-specific reference range, acknowledging inter-assay variability and harmonization challenges. Second, TSH suppression is now stratified more conservatively: values within the reference range are considered appropriate for patients with ER or IndR, whereas values below the reference range are advised for those with BIR or SIR [27]. These refinements reflect a shift toward individualized biochemical monitoring based on assay performance and patient risk dynamics.

Therefore, this study was designed to examine how closely real-world TSH management reflects ATA guideline targets and to quantify the influence of assay-specific reference ranges on risk-adapted TSH modulation after DTC treatment.

## 2. Material and Methods

In our centers, patients with differentiated thyroid carcinoma (DTC) undergo their first post-treatment reassessment 6–12 months after the initial therapy, during which levothyroxine therapy is titrated according to the response-to-therapy classification. Subsequent follow-up evaluations are scheduled 6–12 months thereafter, at the discretion of the treating physician. At each visit, venous blood samples are obtained for routine measurement of TSH, thyroglobulin (Tg), and anti-thyroglobulin antibodies (TgAb). For the purposes of the present study, residual serum material from the second follow-up visit was used (i.e., samples collected ≥12 months after the initial therapy and ≥6 months after response stratification and adjustment of levothyroxine therapy). Thus, one serum specimen per patient was included. Phlebotomy was performed in the morning under fasting conditions. Residual serum aliquots were stored at –80 °C, transported by certified carriers, and centralized for analysis at the Laboratory of Clinical Chemistry, Gruppo Ospedaliero Moncucco/Sonic-Medysin^®^ AG, Lugano, Switzerland. The patients’ disease status (i.e., response to therapy) at the time of blood sampling was centrally assigned by an experienced endocrine oncologist (L.G.) in accordance with the 2015 ATA guidelines, based on a longitudinal review of standardized clinical records. Serum TSH concentrations were measured concurrently using three automated immunoassay platforms: the Elecsys^®^ TSH assay (Roche, Rotkreuz, Switzerland), the Atellica^®^ IM TSH assay (Siemens Healthineers, Zurich, Switzerland), and the Alinity TSH assay (Abbott, Baar, Switzerland). All assays were calibrated, maintained, and operated strictly according to the respective manufacturers’ instructions. Quality-control procedures remained within acceptable limits throughout the study period. The principal analytical characteristics of the three TSH immunoassays are summarized in Table 2.

### Statistical Analysis

Statistical analysis addressed two aims: (i) analytical comparability of TSH measurements across immunoassays, and (ii) clinical appropriateness of TSH suppression relative to guideline targets. The agreement between Elecsys, Atellica, and Alinity was evaluated using paired serum samples, with Passing–Bablok regression (a nonparametric, robust method to outliers) to estimate the slope and intercept, along with 95% confidence intervals. Additionally, Bland–Altman analysis was performed to quantify the mean bias and 95% limits of agreement. An experienced endocrinologist assigned each patient to a response-to-therapy category (ER, IndR, BIR, or SIR) according to the ATA criteria. For each category, we defined a target TSH range according to the ATA 2015 recommendations (ER: 0.5–2.0 mIU/L; IndR/BIR: 0.1–0.5 mIU/L; SIR: <0.1 mIU/L). We then reclassified patients using the ATA 2025 approach, employing assay-specific reference ranges (RRs) (Table 2). ER and IndR patients were considered in-target when TSH was within the platform’s RR, whereas BIR and SIR patients were in-target when TSH was below the platform’s lower reference limit. Accordingly, no category below the target applies to BIR and SIR. For each assay platform and response category, measured TSH was classified as below, within, or above the recommended target range, and the proportion of patients in each stratum was summarized (n and %). To visualize these findings, adherence to guideline targets was displayed as stratified heatmaps, and the overall distribution of TSH values across response categories and assays was displayed as violin plots on a log10 scale, with overlaid guideline target bands. All analyses were performed in R (version 4.4.2. R Core Team (2025). R: A language and environment for statistical computing. R Foundation for Statistical Computing, Vienna, Austria. Available at: https://www.R-project.org/.).

## 3. Results

Serum samples from 220 consecutive patients with DTC were included in this study. Baseline demographic, pathological, and clinical data are summarized in Table 3. The cohort had a slight female predominance (124/220, 56%), and the majority of tumors were papillary thyroid carcinoma (181/220, 82%), followed by follicular thyroid carcinoma (33/220, 15%). Most patients were AJCC stage I or II at presentation (109/220 [50%] stage I, 74/220 [34%] stage II), whereas only 11/220 (5%) were stage IV. At the time of serum sampling, the response-to-therapy status according to ATA categories indicated that 106/220 (48%) patients had ER, 53/220 (24%) had IndR, 31/220 (14%) had BIR, and 30/220 (14%) had SIR. The mean age at sampling was 50.3 ± 20.4 years.

### 3.1. Agreement Between Different TSH Immunoassays

**Figure 1:**
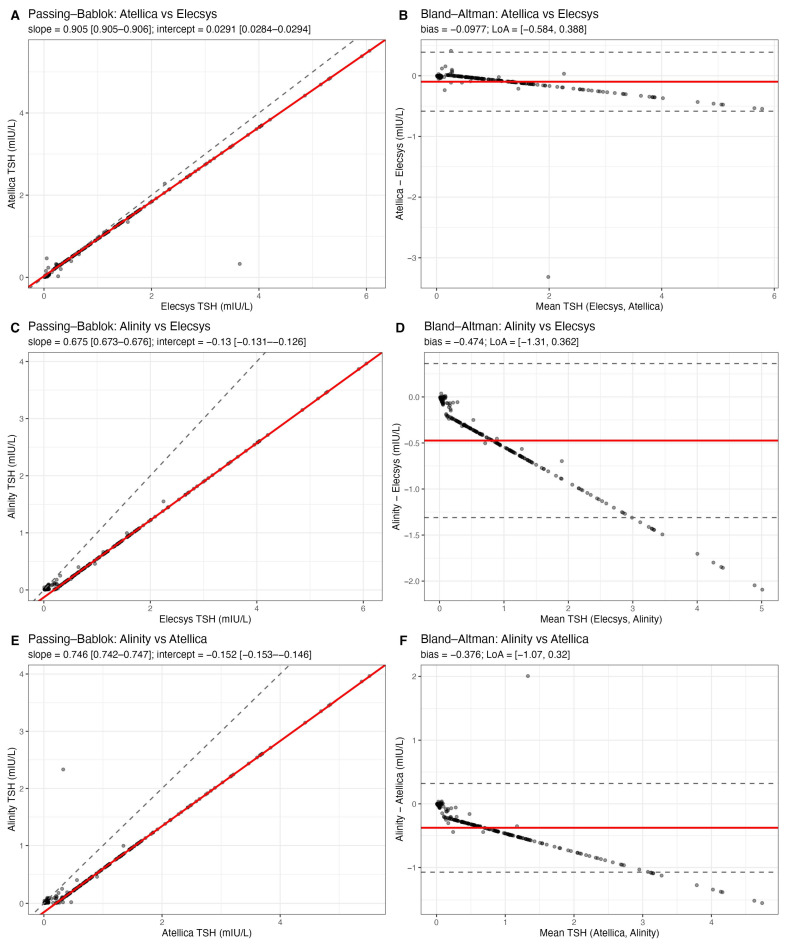
Analytical agreement between TSH immunoassays. Passing–Bablok regression (left) and Bland–Altman plots (right) comparing Atellica vs. Elecsys (top), Alinity vs. Elecsys (middle), and Alinity vs. Atellica (bottom). Dashed lines indicate the line of identity (Passing–Bablok) or the 95% limits of agreement (Bland–Altman); solid red lines indicate the fitted regression or mean bias. Atellica showed minimal proportional bias vs. Elecsys (slope 0.905, intercept 0.029 mIU/L). Alinity showed lower TSH values overall (slope 0.675 vs. Elecsys; bias −0.47 mIU/L), but the magnitude of disagreement remained within clinically acceptable limits.

Pairwise method comparison between the three TSH immunoassays (Elecsys, Atellica, and Alinity) demonstrated high analytical agreement and only minor systematic differences (Figure 1).

Passing–Bablok regression for Atellica vs. Elecsys yielded a slope of 0.905 (95% CI 0.905–0.906) and an intercept of 0.029 mIU/L (95% CI 0.028–0.029), indicating a slight proportional difference but essentially no constant bias. For Alinity vs. Elecsys, the slope was 0.675 (95% CI 0.673–0.676) and the intercept was −0.130 mIU/L (95% CI −0.131 to −0.126), consistent with the slightly lower absolute TSH values reported by Alinity, particularly at higher concentrations. For Alinity vs. Atellica, the slope was 0.746 (95% CI 0.742–0.747) and the intercept was −0.152 mIU/L (95% CI −0.153 to −0.146), again supporting a proportional underestimation by Alinity relative to the comparator assay. Bland–Altman analysis confirmed that these differences were minor in magnitude and unlikely to influence clinical decision-making. The mean bias (Atellica-Elecsys) was −0.098 mIU/L, with 95% limits of agreement from −0.584 to +0.388 mIU/L. The mean bias (Alinity-Elecsys) was −0.474 mIU/L, with a 95% limits of agreement of −1.31 to +0.36 mIU/L. The mean bias (Alinity-Atellica) was −0.376 mIU/L, with a 95% limits of agreement of −1.07 to +0.32 mIU/L. Negative bias values indicate that Atellica and especially Alinity tend to report slightly lower TSH values than Elecsys on average. Notably, even the widest limits of agreement remain well within ranges that would not generally alter levothyroxine management (i.e., they do not move patients across clinically meaningful TSH cutoffs such as 0.1, 0.5, or 2.0 mIU/L in a systematic way). Overall, these data indicate that the three platforms are analytically interchangeable for longitudinal follow-up of DTC patients, and that the variability observed in clinical practice is unlikely to be driven by assay discordance. 

### 3.2. TSH Values Outside Target Ranges by Response Class

**Figure 2:**
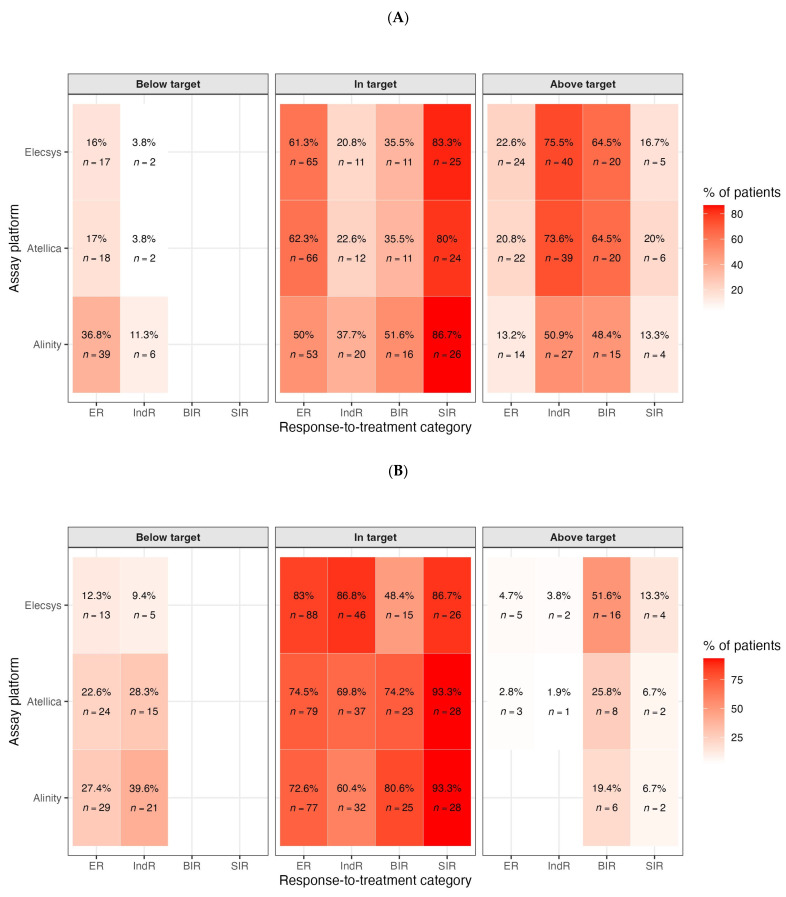
(**A**). Concordance with the ATA 2015 TSH targets. Heatmap showing, for each response-to-therapy category (ER, IndR, BIR, SIR) and assay platform (Elecsys, Atellica, Alinity), the percentage of patients whose TSH was below, within, or above the ATA 2015 recommended range (ER: 0.5–2.0 mIU/L; IndR/BIR: 0.1–0.5 mIU/L; SIR: <0.1 mIU/L). Percentages and absolute counts (n) are reported in each tile. Excellent Response patients were frequently oversuppressed (below target), whereas Indeterminate and Biochemical Incomplete Response patients were commonly under-suppressed (above target). Most Structural Incomplete Response patients were appropriately suppressed. (**B**). Concordance with response-adapted ATA 2025 targets. Heatmap analogous to Figure 2A, applying a response-adapted framework consistent with ATA 2025, which allows de-escalation of TSH suppression in Excellent Responders while maintaining suppression in patients with biochemical or structural persistence. Under this framework, a larger proportion of Excellent Response patients fall “in target,” whereas many Indeterminate Response patients remain above target (undertreated).

We next assessed whether the measured TSH values were consistent with guideline-defined targets. For each patient, and for each assay (Elecsys, Atellica, Alinity), we classified the observed TSH as below, within, or above the recommended range for that patient’s response-to-therapy category. Targets were first defined according to the ATA 2015 guidance: 0.5–2.0 mIU/L for ER, 0.1–0.5 mIU/L for IndR and BIR, and <0.1 mIU/L for SIR. We then repeated the same classification using a response-adapted framework consistent with the evolving ATA 2025 approach, which explicitly allows progressive relaxation of TSH suppression. Under the ATA 2015 targets, we observed marked deviations from guideline recommendations, and the pattern of deviation depended strongly on the clinical response category (Figure 2A).

Among patients with ER, a considerable fraction remained more suppressed than recommended: between 16.0% and 36.8% of ER patients (n = 17–39, depending on assay) had TSH values below 0.5 mIU/L, i.e., below the guideline target range of 0.5–2.0 mIU/L, despite being considered disease-free. This suggests persistent overtreatment and prolonged TSH suppression in a group for whom current practice guidelines generally recommend de-escalation. The opposite phenomenon emerged in patients who did not have an unequivocally excellent response. In IndR, the dominant deviation was undersuppression: up to 75.5% of IndR patients (n = 40) had TSH values above 0.5 mIU/L, i.e., above the recommended range of 0.1–0.5 mIU/L. A similar pattern was observed in the BIR group, where 48.4% to 64.5% of patients (n = 15–20) were abovetarget. In other words, in patients with biochemical or indeterminate evidence of possible residual disease, TSH was often higher than the range recommended for oncologic safety. By contrast, TSH management in patients with SIR, who by definition have persistent structural disease and are expected to remain entirely suppressed, was aligned mainly with guideline expectations: 80.0% to 86.7% of SIR patients (n = 24–26) were within the recommended suppression target (<0.1 mIU/L), and only a minority exceeded 0.1 mIU/L. Using the ATA 2025 assay-specific targets (within the reference range (RR) for ER and IndR, and below the lower reference limit for BIR and SIR), the pattern of apparent non-adherence changed meaningfully (Figure 2B).

**Figure 3:**
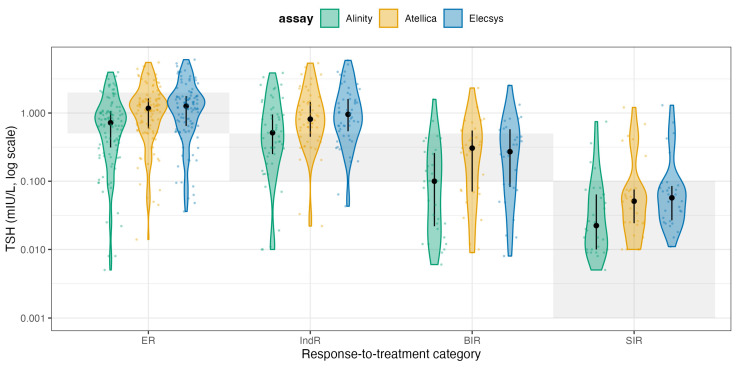
Distribution of TSH relative to guideline targets. Violin plots (log10 scale) showing TSH distributions for each assay (Alinity, Atellica, Elecsys) across response-to-therapy categories. Black points and bars indicate median and IQR. Gray shaded bands represent the guideline target TSH range for that category. ER patients often remain more suppressed than recommended, IndR/BIR patients are frequently under-suppressed, and SIR patients are generally maintained within the intended full-suppression range.

In ER, the in-target share rose to 72.6–83.0% (Alinity 72.6%, Atellica 74.5%, Elecsys 83.0%), with residual over-suppression (i.e., below the RR) in 12.3–27.4% and very few above RI (0–4.7%). In IndR, most patients were also in-target (60.4–86.8%), though a non-trivial fraction remained below the RR (9.4–39.6%), while above-RR cases were rare (0–3.8%). For BIR, where the therapeutic goal is TSH below the assay’s RRs, 48.4–80.6% of patients were in-target, while 19.4–51.6% were above the RR (i.e., below-target cells). Finally, in SIR patients, adherence to complete TSH suppression was high across platforms (86.7–93.3% in-target), with only 6.7–13.3% above RRs. Overall, assay-specific 2025 targets classify a much larger share of ER and IndR patients as appropriately managed, while still highlighting residual undersuppression in a subset of the BIR group and a small minority of SIR patients. To visualize how these effects distribute across the entire TSH spectrum, we plotted violin distributions by response class and assay on a log scale (Figure 3).

For readability, the shaded horizontal bands reflect the fixed-ATA 2015 target ranges for each response category (ER 0.5–2.0 mIU/L; IndR and BIR 0.1–0.5 mIU/L; SIR < 0.1 mIU/L), whereas the ATA 2025 panel applies assay-specific reference intervals. In these violin plots, each shaded horizontal band represents the guideline-recommended TSH range for that specific response category, and the assay-specific TSH distributions (Elecsys, Atellica, Alinity) are overlaid together with the median and interquartile range. These plots make the clinical pattern visually obvious: ER patients often remain below their recommended range (i.e., they are still being aggressively suppressed when guidelines would allow relaxation), IndR and BIR patients frequently sit above their recommended range (i.e., are relatively under-suppressed), and SIR patients cluster tightly in the <0.1 mIU/L region, indicating that clinicians maintain high-intensity suppression primarily in those with clear structural disease. Taken together, these data show two key points. First, Elecsys, Atellica, and Alinity exhibit only modest proportional differences and small absolute biases, supporting their practical interchangeability for TSH monitoring in DTC. Second, when the ATA 2025 assay-specific targets are applied, part of the apparent undertreatment seen under fixed cutoffs is reconciled in ER/IndR. In contrast, a clinically relevant gap persists in BIR with TSH remaining above the intended suppression zone in a non-trivial subset of patients. More importantly, the significant variability in TSH values relative to guideline targets is driven predominantly by prescribing behavior rather than analytical performance. In current practice, many Excellent Responders remain more suppressed than necessary, whereas patients with only indeterminate or biochemical evidence of residual disease are often less suppressed than recommended. Applying a response-adapted framework consistent with ATA 2025 reclassifies a substantial proportion of this “non-adherence” among Excellent Responders as acceptable, individualized de-escalation, yet still reveals a clinically relevant gap in TSH suppression among patients without a clearly Excellent Response.

## 4. Discussion

The present analysis highlights the persistent gap between guideline-recommended and real-world TSH management in patients with DTC [28]. Although the 2015 ATA guidelines proposed distinct TSH suppression targets based on recurrence risk and response to therapy, multiple observational studies and registry data indicate that these targets are inconsistently achieved in clinical practice. This variability reflects both physician-driven decisions—often balancing oncologic safety with comorbidity risk—and analytical differences among TSH assays, which complicate the uniform application of targets. Additionally, recent evidence challenges the long-standing assumption that profound TSH suppression (≤0.1 mIU/L) uniformly improves prognosis [29]. A large multicenter cohort and meta-analyses demonstrate that, among low- and intermediate-risk patients, maintaining TSH within or moderately below the reference range does not increase the risk of recurrence or mortality [30]. Conversely, persistent suppression below the physiological range has been associated with a higher incidence of atrial fibrillation, bone mineral loss, and impaired quality of life [31]. These findings reinforce the rationale for the recent 2025 ATA guideline update, which redefines TSH goals relative to the assay-specific reference range rather than absolute fixed cutoffs. In this context, our comparison of three widely used immunoassay platforms—Elecsys, Atellica, and Alinity—demonstrated a very high degree of analytical alignment, with minimal systematic bias and strong correlation across the clinically relevant TSH range. Our results perfectly align with those recently reported by Ursem and colleagues. They compared Elecsys, Alinity, and Atellica assays in the low TSH range (<0.4 mIU/L) and reported minor imprecision (1–14%) with negligible clinical impact [32]. This concordance supports the reliability of cross-platform monitoring in most clinical scenarios. It suggests that assay variability may be less influential than previously assumed when high-quality, standardized platforms are used. In turn, the good agreement between assays reflects improvements in TSH assay comparability resulting from the use of traceable, commutable reference materials and calibration traceability, as proposed by the International Federation of Clinical Chemistry (IFCC) Working Group for Standardization of Thyroid Function Tests [33,34]. Nevertheless, minor differences near the lower reference limit may still impact clinical interpretation in patients requiring tight TSH control, underscoring the importance of maintaining awareness of platform-related nuances. The shift from absolute numerical thresholds to reference-based targets represents a significant conceptual advance. It acknowledges both residual inter-assay variability and the need for patient-specific interpretation of biochemical data. Future strategies should emphasize assay harmonization, individualized monitoring, and dynamic risk-adapted titration. Laboratories and clinicians should consistently report TSH values together with their reference ranges and ensure internal traceability to international standards. Clinically, suppression intensity should be reassessed over time as the disease status evolves. For patients with an excellent or indeterminate response, maintaining TSH within the normal range appears both safe and desirable. In contrast, suppression below the reference limit may remain appropriate for biochemical or structural incomplete responses. These new concepts should be clearly explained to our patients, especially to those who have been on long-term suppressive therapy and may therefore be reluctant to modify their treatment regimen. A calm and balanced presentation of risks and benefits—while also considering the psychological implications—represents the key to successfully introducing these new paradigms into clinical practice. Prospective multicentric studies integrating biochemical and clinical parameters are warranted to refine and validate this more flexible, response-driven approach.

## 5. Conclusions

In summary, our findings confirm that TSH values in patients treated for DTC are generally consistent across modern immunoassay platforms, supporting reliable biochemical follow-up. However, real-world TSH levels often deviate from traditional suppression targets, reflecting an evolving balance between oncologic control and the prevention of iatrogenic harm. The updated 2025 ATA guidelines acknowledge these realities and shift from fixed numerical thresholds to assay-specific ranges, providing a more flexible, physiologic framework that balances oncologic control with long-term safety. The routine integration of assay-specific reference data and dynamic risk stratification will be essential to refine the management of patients with DTC.

## Figures and Tables

**Table 1:** Differentiated thyroid carcinoma: definitions or response categories, risk of recurrence, and target TSH ranges.

Response	Criteria	AdRR	TSH Target ATA 2015	TSH Target ATA 2025
ER	Imaging: negative onT4-Tg <0.2 ug/L *OR* Stim-Tg <1 ug/L *AND* negative TgAb	<5%	0.5–2.0 mIU/L	within the RR
IndR	imaging: nonspecific findings onT4-Tg 0.2–1 ug/L *OR* Stim-Tg 1–10 ug/L *OR* stable/declining TgAb	15–20%	0.1–0.5 mIU/L	within the RR
BIR	Imaging: no evidence of disease. onT4-Tg >1 ug/L *OR* Stim-Tg >10 ng/mL *OR* rising TgAb	~20%	0.1–0.5 mIU/L	below the RR
SIR	Imaging: persistent or new locoregional or distant metastases Any Tg/TgAb value	50–85%	<0.1 mIU/L	below the RR

Legend: ER, excellent response; IndR, indeterminate response; BIR, biochemical incomplete response; SIR, structural incomplete response; Tg, thyroglobulin; TgAb, thyroglobulin antibodies; onT4-Tg, on thyroxine-Tg; Stim-Tg, stimulated-Tg; imaging (US, ultrasound; CT, computed tomography; PET/CT, positron-emission tomography/CT); AdRR, adjusted risk of recurrence.

**Table 2:** Analytical characteristics of different TSH immunoassays employed in our study.

Assays	Manufacturer	Technology	LoD	LoQ	Measuring Range (mIU/L)	RR (mIU/L)
Elecsys	Roche	ECLIA	0.005	0.005	0.005–100	0.27–4.20
Atellica	Siemens	CLIA	0.003	0.004	0.004–100	0.55–4.78
Alinity	Abbott	CMIA	0.007	0.01	0.01–100	0.35–4.94

Legend: ECLIA, electrochemiluminescence immunoassay; CLIA, chemiluminescent immunoassay; CMIA, chemiluminescent microparticle immunoassay; LoD, Limit of Detection; LoQ, Limit of Quantitation; RI, reference range.

**Table 3:** Demographic, clinical, and pathological data, and treatment response categories.

	n (%)
Total patients	220
Female	124 (56%)
Male	96 (43%)
Age (years)	50.3 ± 20.40
DTC histotypes	220
PTC	181 (82%)
FTC	33 (15%)
Others	6 (3%)
Stage (AJCC)	
I	109 (50%)
II	74 (34%)
III	26 (11%)
IV	11 (5%)
Response to treatment (ATA)	
ER	106 (48%)
IndR	53 (24%)
BIR	31 (14%)
SIR	30 (14%)

Legend: DTC, differentiated thyroid cancer; PTC, papillary thyroid cancer; FTC, follicular thyroid cancer; AJCC, American Joint Committee on Cancer; ATA, American Thyroid Association; ER, excellent response; IndR, indeterminate response; BIR, biochemical incomplete response; SIR, structural incomplete response.

## Data Availability

For information on the study and data sharing, qualified researchers may contact the corresponding author, Luca Giovanella (luca.giovanella@moncucco.ch).
